# Mental health of people living with hiv and adherence to antiretroviral therapy

**DOI:** 10.1192/j.eurpsy.2021.656

**Published:** 2021-08-13

**Authors:** F. Goma, G. Papazisis, E. Papakonstantinou, G. Karakioulakis

**Affiliations:** 1 1st Department Of Pharmacology, School of Medicine, Aristotle University of Thessaloniki, Thessaloniki, Greece; 2 Department Of Clinical Pharmacology, School of Medicine, Aristotle University of Thessaloniki, THESSALONIKI, Greece

**Keywords:** HIV, mental health, adherence, antiretroviral therapy

## Abstract

**Introduction:**

Adherence to antiretroviral therapy is a key factor in predicting the success or failure of treatment. Data suggest that the status of mental health and especially depression of people living with HIV can affect adherence to antiretroviral therapy.

**Objectives:**

The purpose of this study was to assess the mental health status of people living with HIV, to record adherence to antiretroviral therapy and to investigate whether mental health affects adherence to antiretroviral therapy.

**Methods:**

A cross-sectional mixed observational correlation study in a sample of 112 HIV-positive individuals was conducted. The Simplified Medication Adherence Questionnaire (SMAQ) was used to assess adherence to antiretroviral therapy, the Beck Depression Inventory (BDI) was used to assess depression, and the WHOQOL – BREF tool was used to assess mental health.

**Results:**

The results of the study showed that 58.93% of patients were found to be non-adherent to antiretroviral therapy. Furthermore, according to the BDI scale, 10.7% of patients experienced marginal clinical depression, 10.7% experienced moderate levels of depression and 2.7% experienced severe or very severe levels of depression. Further, people living with HIV had a moderate level of mental health (M = 3.40, SD = 0.58).

**Conclusions:**

Our study showed that a high percentage of people living with HIV are non-adherent to antiretroviral therapy. Factors that are possibly associated with decreased adherence are mental health and especially depression. Psychological support for people living with HIV and anti-depressant prevention programs could increase adherence to antiretroviral therapy.

